# Chemical composition and insecticidal activity of plant essential oils from Benin against *Anopheles gambiae* (Giles)

**DOI:** 10.1186/1756-3305-6-337

**Published:** 2013-12-03

**Authors:** Annick D Bossou, Sven Mangelinckx, Hounnankpon Yedomonhan, Pelagie M Boko, Martin C Akogbeto, Norbert De Kimpe, Félicien Avlessi, Dominique C K Sohounhloue

**Affiliations:** 1 Laboratoire d’Etude et de Recherche en Chimie Appliquée, Ecole Polytechnique d’Abomey-Calavi, Université d’Abomey-Calavi, Cotonou 01 BP 2009, Bénin; 2 Department of Sustainable Organic Chemistry and Technology, Faculty of Bioscience Engineering, Ghent University, Coupure links 653, Ghent B-9000, Belgium; 3 Herbier National du Bénin, Laboratoire de Botanique et Ecologie Végétale, Université d'Abomey-Calavi, Cotonou 01 BP 4521, Bénin; 4 Centre de Recherche en Entomologie de Cotonou, Cotonou 06 BP 2604, Bénin

**Keywords:** Malaria, *A. gambiae*, Essential oils, Diagnostic dose, Knock-down times, Insecticide, Benin

## Abstract

**Background:**

Insecticide resistance in sub-Saharan Africa and especially in Benin is a major public health issue hindering the control of the malaria vectors*.* Each *Anopheles* species has developed a resistance to one or several classes of the insecticides currently in use in the field. Therefore, it is urgent to find alternative compounds to conquer the vector. In this study, the efficacies of essential oils of nine plant species, which are traditionally used to avoid mosquito bites in Benin, were investigated.

**Methods:**

Essential oils of *nine plant species* were extracted by hydrodistillation, and their chemical compositions were identified by GC-MS. These oils were tested on susceptible “*kisumu*” and resistant “*ladji-*Cotonou” strains of *Anopheles gambiae*, following WHO test procedures for insecticide resistance monitoring in malaria vector mosquitoes*.*

**Results:**

Different chemical compositions were obtained from the essential oils of the plant species. The major constituents identified were as follows: neral and geranial for *Cymbopogon citratus*, *Z*-carveol, *E*-*p*-mentha-1(7),8-dien-2-ol and *E*-*p*-mentha-2,8-dienol for *Cymbopogon giganteus*, piperitone for *Cymbopogon schoenanthus*, citronellal and citronellol for *Eucalyptus citriodora*, *p*-cymene, caryophyllene oxide and spathulenol for *Eucalyptus tereticornis*, 3-tetradecanone for *Cochlospermum tinctorium* and *Cochlospermum planchonii*, methyl salicylate for *Securidaca longepedunculata* and ascaridole for *Chenopodium ambrosioides*. The diagnostic dose was 0.77% for *C. citratus,* 2.80% for *E. tereticornis*, 3.37% for *E. citriodora*, 4.26% for *C. ambrosioides*, 5.48% for *C. schoenanthus* and 7.36% for *C. giganteus.* The highest diagnostic doses were obtained with *S. longepedunculata* (9.84%), *C. tinctorium* (11.56%) and *C. planchonii* (15.22%), compared to permethrin 0.75%. *A. gambiae* cotonou, which is resistant to pyrethroids, showed significant tolerance to essential oils from *C. tinctorium* and *S. longepedunculata* as expected but was highly susceptible to all the other essential oils at the diagnostic dose.

**Conclusions:**

*C. citratus*, *E. tereticornis*, *E. citriodora, C. ambrosioides* and *C. schoenanthus* are potential promising plant sources for alternative compounds to pyrethroids, for the control of the *Anopheles* malaria vector in Benin. The efficacy of their essential oils is possibly based on their chemical compositions in which major and/or minor compounds have reported insecticidal activities on various pests and disease vectors such as *Anopheles*.

## Background

Malaria is a life-threatening disease affecting 3.3 billion people worldwide with 80% of cases and 90% of deaths occurring in sub-Saharan Africa (SSA). Of those affected, children under the age of five and pregnant women are the most vulnerable [1]. The key factor for the reduction of human mortality is their protection against the malaria parasite vectors, the *Anopheles* mosquito, by the use of long-lasting insecticidal nets (LLIN) and application of indoor residual spraying (IRS) as recommended by the WHO [1]. Pyrethroids are the only insecticides recommended by WHO for the treatment of bednets [1] while in West Africa, *Anopheles gambiae* Giles is the major vector of malaria [2]. This mosquito species is now subjected to selection pressure, and has become resistant to dichlorodiphenyltrichloroethane (DDT) and pyrethroids because of the use of these insecticides both for agricultural and public health purposes [2,3]. During the last decade, the emergence of resistance to synthetic insecticides in *Anopheles* populations has been widely described in many African countries such as Benin [4-10], Ivory Coast [2,11], Niger [12], Burkina Faso [3,13,14], Mali [15], Nigeria [16], Kenya [17], Cameroun [18-20], Zanzibar [21], Uganda [22], Equatorial Guinea [23], and Ghana [24]. Because of the widespread occurrence of this resistance, there is a reduction of the efficacy of synthetic insecticides used in the field. Therefore, it is pertinent to explore the pesticidal activity of natural products [25] such as essential oils. Indeed the use of essential oils has been recognized as a potential alternative in the control of vectors of mosquito-borne diseases [26-30] such as *A. gambiae*. A lot of research on the pesticidal activities of essential oils has been conducted and has proven that essential oils could be considered as potent bioactive compounds against various pests and mosquitoes [28,31-34].

The objective of the current study was to investigate the chemical composition and the insecticidal activities of essential oils of nine aromatic plants traditionally used in Benin as a natural method of protection against *A. gambiae*, in order to confirm the traditional knowledge of Benin’s population and to find new valuable sources of active molecules against this malaria vector*.* Indeed these plant species have been reported to possess repellent and insecticidal activities against various mosquitoes, stored product beetles and other pests such as *Anopheles* species [30,35]*, Culex quinquefasciatus*[36], *Callosobruchus* species [37,38], *Sitophilus* species [39,40] and *Tribolium castaneum*[41]. These plants include *Chenopodium ambrosioides* L. (Amaranthaceae), *Securidaca longepedunculata* Fresen. (Polygalaceae), *Cochlospermum planchonii* Hook. f. Ex Planch. (Bixaceae), *Cochlospermum tinctorium* A. Rich. (Bixaceae), *Eucalyptus tereticornis* Sm. (Myrtaceae), *Eucalyptus citriodora* Hook. (Myrtaceae), *Cymbopogon citratus* (DC.) Stapf (Poaceae), *Cymbopogon schoenanthus* (L.) Spreng. (Poaceae) and *Cymbopogon giganteus* Chiov. (Poaceae).

For this purpose, the standard WHO susceptibility test has been used with various concentrations of essential oils under laboratory conditions [42].

## Methods

### Plant material

Nine plant species belonging to five botanical families were collected in several regions of Benin. Collected plant material was dried, free from light, at room temperature. All these plants have been the subject of classification and botanic description at the National Herbarium of the University of Abomey-Calavi, Benin [43]. Locations of collection, organs extracted and nature of soil are summarized in Table 1.

**Table 1:** Locations, soils and organs extracted from selected plants species

**Plants species**	***Cymbopogon citratus***	***Cymbopogon giganteus***	***Cymbopogon schoenanthus***	***Cochlospermum planchonii***	***Cochlospermum tinctorium***	***Eucalyptus citriodora***	***Eucalyptus tereticornis***	***Chenopodium ambrosioides***	***Securidaca longepedunculata***
Locations	Cotonou	Koudo	Nalohou 2	Mount Kassa	Mount Kassa	Abomey Calavi	Abomey Calavi	Savalou	Savalou
Soils	Sandy	Ferralitic	Gravelly ferruginous	Ferruginous	Ferruginous	Ferralitic	Ferralitic	Ferruginous	Ferruginous
Organs	Leaves	Leafy stems	Leafy stems	Roots	Roots	Leaves	Leaves	Leafy stems	Root bark
Certification numbers	AA6463/HNB	AA6464/HNB	AA6465/HNB	AA6466/HNB	AA6467/HNB	AA6468/HNB	AA6469/HNB	AA6470/HNB	AA6471/HNB

### Essential oils

Essential oils from all plants were extracted by hydrodistillation using a Clevenger-type apparatus for two to four hours until essential oils were extracted completely from the organs. The extracted oils were dried over anhydrous magnesium sulfate and stored at 4°C, away from UV rays, before use. Each essential oil was diluted tenfold with diethyl ether. One milliliter of each diluted solution was charged into a sampler flask for GC-MS analysis.

### Chemical analyses of essential oils by gas chromatography coupled with mass spectrometry

The GC-MS analysis of the essential oils was performed on an Agilent 6890 GC Plus automatic sampler system, coupled to a quadruple mass spectrometer 5973 MSD (Agilent Technologies, Diegem, Belgium) and equipped with a capillary HP-5MS column fused with silica (length: 30 m; diameter: 0.25 mm, film thickness: 0.25 microns) in the split mode 1:100. The oven temperature was programmed at 60°C for 3 min and to 350°C at a rate of 5°C/min. The injector was kept at 250°C programmed with a rate of 10°C/s. Helium was used as carrier gas at a flow rate of 1 ml/min. All analyses were performed at constant flow. The mass detector conditions were: transfer line temperature 260°C; ionization mode electron impact: 70 eV. The identification of sample compounds was carried out in single runs. The Kovats retention indices were calculated for all volatile constituents using a series of n-alkanes C_7_-C_17_[44]. Quantification of each compound was performed using percentage peak area calculations. The identification of oil volatile compounds was done by comparing their retention indices with those of reference compounds in the literature and confirmed by GC-MS by comparison of their mass spectra with those of reference compounds [44-47]. The relative concentration of each compound in the essential oil was quantified according to the peak area integrated by the analysis program (Chemstation data analysis).

### Mosquito collection

Larvae of the resistant strain of *A. gambiae* were collected three times a week, from March 2012 to June 2012, from the natural breeding site in “Ladji” in Cotonou. Ladji is a neighborhood of the outskirts of Cotonou, the capital of Benin. Larvae were brought and reared in the insectary of the Cotonou Research Center of Entomology (CREC). Larvae were fed with honey juice and the adults were placed in cages and fed in the same way. Emerging adult female mosquitoes of the resistant strain were used to carry out the susceptibility tests, whereas a susceptible strain of *A. gambiae “Kisumu”* originating from Kenya and maintained at the insectary, were used as a reference.

### Bioassay on adult mosquitoes

Susceptibility tests were carried out using WHO insecticide susceptibility test-kits and standard procedures [42]. Four replicates of batches of 20-25 unfed females, aged 2-5 days old were exposed to filter papers (Whatman N°1) 12 cm × 15 cm, impregnated with 2 ml of various doses of the essential oil, diluted in acetone. The susceptible strain *A. gambiae “Kisumu”* was used as reference to determine the diagnostic doses. The filter papers of the control holding tubes were impregnated with acetone only. Tests were carried out at 25°C (± 2°C) and 70-80% relative humidity. Six doses were tested for each essential oil: 0.25% (w/v), 0.50% (w/v), 1% (w/v), 2% (w/v), 4% (w/v) and 8% (w/v). The number of mosquitoes knocked down was recorded every 5 min. Permethrin 0.75% was used as reference insecticide. Permethrin impregnated papers were obtained from the WHO reference center of “the Vector Control Research Unit of The University Sains Malaysia”. After the exposure time, mosquitoes were transferred to holding tubes and were fed with 10% honey juice for 24 hours. Subsequently the mortality was recorded. Data were plotted to determine the diagnostic doses to which the resistant strain from Ladji was exposed.

### Data analysis for bioassay

Times at which 50% or 95% of mosquitoes fell down on their back or on their side, i.e. knockdown time (KDT_50_ and KDT_95_) was calculated by means of the log time-probit using SPSS 17.0 software. The relation between the KDT, the mortality and the doses were assessed by probit regression. The diagnostic concentration which is the double of the minimum concentration at which 100% of the susceptible strain died [48] was also taken into account. All the results obtained for KDT (KDT_50_ and KDT_95_) and lethal doses (LC_50_ and LC_99_), were expressed with 95% confidence limits.

## Results and discussion

### Chemical composition of essential oils

Chemical compositions and essential oil yields, expressed as oil wt./wt. of dried organ extracted, showed a large variation (Tables 2, 3, 4, 5 and 6). These yields varied from 0.2% for *Cochlospermum* species to 4.6% for *E. citriodora*.

**Table 2:** **Chemical composition and oil yields of *Cymbopogon *** **species**

**KI**_**exp**_^**a**^	**KI**_**lit**_^**b**^	**ID**^**c**^	**Compounds**^**d**^	***Cymbopogon citratus***	***Cymbopogon giganteus***	***Cymbopogon schoenanthus***
				***%***	***%***	***%***
978	974	MS, RI	Sulcatone	0.5		
982	988	MS, RI	**Myrcene**	**12.4**		
984	988	MS, RI	2,3-Dehydro-1,8-cineole		0.1	0.1
995	1001	MS, RI	**δ-2-Carene**		0.1	**15.5**
998	1002	MS, RI	α-Phellandrene			0.2
1009	1014	MS, RI	α-Terpinene			0.2
1017	1020	MS, RI	*p*-Cymene		0.5	0.1
1020	1024	MS, RI	**Limonene**		**9.6**	3.6
1029	1032	MS, RI	*Z*-β-Ocimene	0.3		0.1
1039	1044	MS, RI	*E*-β-Ocimene	0.2		0.1
1081	1083	MS, RI	L-Fenchone			0.2
1082	1082	MS, RI	*m*-Cymenene		0.2	
1085	1090	MS, RI	6,7-Epoxymyrcene	0.2		
1093	1095	MS, RI	Linalool	1.1		
1114	1118	MS, RI	*Z*-*p*-menth-2-en-1-ol			1.4
1116	1119	MS, RI	***E*- *p *-Mentha-2,8-dienol**		**19.3**	
1129	1133	MS, RI	***Z*- *p *-Mentha-2,8-dienol**		**10.2**	
1132	1136	MS, RI	*E*-*p*-menth-2-en-1-ol			0.7
1138	1144	MS, RI	Neo-Isopulegol		0.1	
1145	1148	MS, RI	Citronellal	0.1	0.7	
1148		MS	4-Isopropenylcyclohexanone		0.4	
1160	1166	MS, RI	*p*-mentha-1,5-dien-8-ol			0.1
1168	1173	MS, RI	Rose furan epoxide	0.2		
1180	1179	MS, RI	*p*-Cymen-8-ol			0.1
1183	1186	MS, RI	α-Terpineol			1.5
1183	1187	MS, RI	***E*-p-Mentha-1(7),8-dien-2-ol**		**19.6**	
1188	1195	MS, RI	*Z*-Piperitol			0.3
1194		MS	*p*-Menth-6-en-2,3-diol		3.2	
1201	1209	MS, RI	*E*-Piperitol			0.2
1212	1215	MS, RI	*E*-Carveol		6.0	
1216	1224	MS, RI	2,3-Epoxyneral	0.1		
1224	1226	MS, RI	***Z*-Carveol**		**17.0**	
1225	1227	MS, RI	*Z*-*p*-Mentha-1(7),8-dien-2-ol		2.1	
1237	1235	MS, RI	**Neral**	**33.1**		
1237	1239	MS, RI	Carvone		3.2	
1253	1249	MS, RI	**Piperitone**		0.1	**58.9**
1257	1249	MS, RI	Geraniol	1.0		
1266	1269	MS, RI	Perilla aldehyde		0.8	
1268	1264	MS, RI	**Geranial**	**44.3**		
1286	1293	MS, RI	2-Undecanone	0.1		
1351	1359	MS, RI	Geranic acid	0.1		
1376	1379	MS, RI	Geranyl acetate	0.8		
1382	1389	MS, RI	β-Elemene			0.4
1408	1417	MS, RI	*E*-Caryophyllene	0.1		1.1
1421	1431	MS, RI	β-Gurjunene			0.1
1443	1452	MS, RI	α-Humulene			0.1
1486	1495	MS, RI	2-Tridecanone	0.1		
1490	1500	MS, RI	α-Muurolene			0.1
1503	1513	MS, RI	γ-Cadinene			0.1
1513	1522	MS, RI	δ-Cadinene			0.4
1540	1546	MS, RI	Elemol			5.3
1571	1582	MS, RI	Caryophyllene oxide	0.1	0.1	
1595	1607	MS, RI	5-Epi-7-epi-α-eudesmol			0.3
1609	1622	MS, RI	10-Epi-γ-eudesmol			0.2
1609	1615	MS, RI	Selina-6-en-4-ol	0.4		
1636	1640	MS, RI	Phenyl ethyl hexanoate		0.1	
1623	1630	MS, RI	γ-Eudesmol			1.1
1626	1629	MS, RI	Eremoligenol			1.9
1634	1640	MS, RI	Hinesol			0.7
1643	1649	MS, RI	β-Eudesmol			1.2
1646	1652	MS, RI	α-Eudesmol			2.1
			Yields	1.7	1.4	2.6
			Total identified	95.2	93.4	98.4

^a^KI_exp_ = retention indices are determined using n-alkanes (C_7_-C_17_).

^b^KI_lit_ = retention indices of reference compounds from literature.

^c^ID = Identification methods; MS = comparison of the mass spectrum with those of the computer mass libraries, and Adams (2007); RI = comparison of calculated RI with those reported in the literature.

^d^Compounds are listed in order of their retention time; the names and the percentages of main compounds are indicated in bold.

**Table 3:** **Chemical composition and oil yields of *Eucalyptus *** **species**

**KI**_**exp**_^**a**^	**KI**_**lit**_^**b**^	**ID**^**c**^	**Compounds**^**d**^	***E. citriodora***	***E. tereticornis***
				**%**	**%**
918	924	MS, RI	α-Thujene		1.7
925	925	MS, RI	α-Pinene	0.1	0.7
964	969	MS, RI	Sabinene		0.5
968	974	MS, RI	β-Pinene	0.2	0.1
982	988	MS, RI	Myrcene	0.1	0.3
998	1002	MS, RI	α-Phellandrene		0.5
1009	1014	MS, RI	α-Terpinene	0.1	0.5
1017	1020	MS, RI	***p*-Cymene**	0.1	**16.7**
1020	1024	MS, RI	Limonene	0.2	
1021	1025	MS, RI	β-Phellandrene		2.9
1023	1026	MS, RI	1,8-Cineole (Eucalyptol)	1.5	2.2
1045	1044	MS, RI	2,6-dimethyl-5-Heptenal	0.2	
1050	1054	MS, RI	γ-Terpinene	0.1	0.7
1080	1086	MS, RI	Terpinolene		0.1
1081	1089	MS, RI	*p*-Cymenene		0.3
1093	1095	MS, RI	Linalool	0.4	0.1
1104	1106	MS, RI	*Z*-Rose oxide	0.1	
1109	1112	MS, RI	*E*-Thujone		0.2
1114	1118	MS, RI	*Z*-*p*-Menth-2-en-1-ol		0.5
1132	1136	MS, RI	*E*-*p*-Menth-2-en-1-ol		0.4
1139	1144	MS, RI	**Neo-Isopulegol**	**7.8**	
1150	1148	MS, RI	**Citronellal**	**52.8**	0.1
1150	1154	MS, RI	Sabina ketone		0.1
1152	1155	MS, RI	Iso-Isopulegol	3.0	
1166	1167	MS, RI	Umbellulone		0.1
1170	1174	MS, RI	Terpinen-4-ol		4.4
1181	1183	MS, RI	**Cryptone**		**11.4**
1184	1186	MS, RI	α-Terpineol	0.3	0.5
1200	1192	MS, RI	α-Phellandrene Epoxide		0.2
1223	1224	MS, RI	*m*-Cumenol		0.5
1224	1223	MS, RI	**Citronellol**	**20.0**	
1232	1238	MS, RI	Cumin aldehyde		3.1
1236	1239	MS, RI	Carvone		0.1
1239	1244	MS, RI	Carvotanacetone		0.1
1246	1249	MS, RI	Piperitone		0.2
1267	1273	MS, RI	*p*-Menth-1-en-7-al		4.2
1268	1274	MS, RI	Neo-isopulegyl acetate	0.3	
1276	1283	MS, RI	α-Terpinen-7-al		0.3
1296	1298	MS, RI	Carvacrol		1.4
1301	1308	MS, RI	*p*-Cymen-7-ol		0.3
1326	1330	MS, RI	3-Oxo-*p*-menth-1-en-7-al		0.2
1346	1350	MS, RI	**Citronellyl acetate**	**9.0**	
1382	1389	MS, RI	β-Elemene		0.1
1391	1392	MS, RI	*Z*-Jasmone	0.2	
1408	1417	MS, RI	*E*-Caryophyllene	0.4	0.5
1443	1452	MS, RI	α-Humulene		0.3
1450	1458	MS, RI	Allo-Aromadendrene		0.9
1485	1488	MS, RI	Bicyclogermacrene	0.1	
1513	1521	MS, RI	*E*-Calamenene		0.2
1568	1571	MS, RI	**Spathulenol**	0.1	**13.5**
1574	1582	MS, RI	**Caryophyllene oxide**		**14.2**
1592	1590	MS, RI	Globulol		0.6
1598	1600	MS, RI	Humulene epoxide II		2.2
1631	1631	MS, RI	Isospathulenol		1.6
1643	1639	MS, RI	Caryophylla-4(12),8(13)-dien-5-β-ol		0.9
1648	1652	MS, RI	α-Cadinol		0.3
1729	1733	MS, RI	Isobicyclogermacrenal		0.8
1753	1759	MS, RI	Cyclocolorenone		0.6
			Yields	4.6	1.0
			Total identified	97.1	92.3

^a^KI_exp_ = retention indices are determined using n-alkanes (C_7_-C_17_).

^b^KI_lit_ = retention indices of reference compounds from literature.

^c^ID = Identification methods; MS = comparison of the mass spectrum with those of the computer mass libraries, and Adams (2007); RI = comparison of calculated RI with those reported in the literature.

^d^Compounds are listed in order of their retention time; the names and the percentages of main compounds are indicated in bold.

**Table 4:** **Chemical composition and oil yields of *Cochlospermum *** **species**

**KI**_**exp**_^**a**^	**KI**_**lit**_^**b**^	**ID**^**c**^	**Compounds**^**d**^	***C. planchonii***	***C. tinctorium***
				***%***	***%***
1025	1020	MS, RI	*p*-Cymene	0.3	
1100	1099	MS, RI	Undecane	0.1	
1154	1148	MS, RI	Citronellal	0.2	
1183	1178	MS, RI	Naphthalene	0.1	
1258	1249	MS, RI	Piperitone	0.1	
1309	1305	MS, RI	Undecanal	0.2	
1382	1389	MS, RI	β-Elemene	1.0	0.6
1388	1398	MS, RI	Cyperene	0.1	0.1
1400	1408	MS, RI	Dodecanal	0.1	0.1
1405	1411	MS, RI	*Z*-α-Bergamotene		0.1
1408	1417	MS, RI	*E*-Caryophyllene		0.1
1425	1432	MS, RI	*E*-α-Bergamotene		0.6
1437	1445	MS, RI	Epi-β-Santalene		0.1
1492	1469	MS, RI	1-Dodecanol	0.5	
1493	1506	MS, RI	*Z*-α-Bisabolene		0.3
1499	1505	MS, RI	β-Bisabolene		2.2
1500	1499	MS, RI	2-Tridecanone	6.8	3.4
1505	1514	MS, RI	*Z*-γ-Bisabolene		0.1
1512	1509	MS, RI	Tridecanal	0.3	
1513	1511	MS, RI	δ-Amorphene		0.1
1515	1524	MS, RI	Methyl dodecanoate		0.5
1522	1529	MS, RI	*E*-γ-Bisabolene		0.3
1578	1576	MS, RI	Dodecanoic acid	1.2	
1576	1574	MS, RI	**Cyclododecanone**		**7.8**
1585	1582	MS, RI	Caryophyllene oxide	0.3	0.1
1598		MS	**3-Tetradecanone**	**24.7**	**48.3**
1599	1607	MS, RI	Dodecyl acetate	4.9	2.0
1602	1611	MS, RI	Tetradecanal		0.3
1663	1658	MS, RI	Neo-Intermedeol	0.1	
1680	1685	MS, RI	α-Bisabolol		0.1
1695	1671	MS, RI	n-Tetradecanol	3.0	
1702	1697	MS, RI	2-Pentadecanone	5.7	0.7
1717	1722	MS, RI	Methyl tetradecanoate		2.3
1775	1780	MS, RI	Tetradecanoic acid	0.5	
1786		MS	**3-Hexadecanone**	2.0	**7.4**
1795	**1795**	MS, RI	**Ethyl tetradecanoate**	**11.4**	
1798		MS	1-Tetradecanyl acetate		4.3
1818	1822	MS, RI	Hexadecanal		0.6
1846	1844	MS, RI	**Isoamyl dodecanoate**	**14.1**	
1874		MS	Pentadecanol	2.0	
2003		MS	4-Octadecanone	2.2	
			Yields	0.2	0.2
			Total identified	81.9	82.5

^a^KI_exp_ = retention indices are determined using n-alkanes (C_7_-C_17_).

^b^KI_lit_ = retention indices of reference compounds from literature.

^c^ID = Identification methods; MS = comparison of the mass spectrum with those of the computer mass libraries, and Adams (2007); RI = comparison of calculated RI with those reported in the literature.

^d^Compounds are listed in order of their retention time; the names and the percentages of main compounds are indicated in bold.

**Table 5:** **Chemical composition and oil yield of *Securidaca longepedunculata***

**KI**_**exp**_^**a**^	**KI**_**lit**_^**b**^	**ID**^**c**^	**Compounds**^**d**^	**%**
1194	1190	MS, RI	**Methyl salicylate**	**99.4**
1439		MS	Methyl 4-methoxysalicylate	0.5
			Yield	0.7
			Total identified	99.9

^a^KI_exp_ = retention indices are determined using n-alkanes (C_7_-C_17_).

^b^KI_lit_ = retention indices of reference compounds from literature.

^c^ID = Identification methods; MS = comparison of the mass spectrum with those of the computer mass libraries, and Adams (2007); RI = comparison of calculated RI with those reported in the literature.

^d^Compounds are listed in order of their retention time: the names and the percentages of main compounds are indicated in bold.

**Table 6:** **Chemical composition and oil yield of *Chenopodium ambrosioides***

**KI**_**exp**_^**a**^	**KI**_**lit**_^**b**^	**ID**^**c**^	**Compounds**^**d**^	**%**
1010	1014	MS, RI	α-Terpinene	16.5
1018	1020	MS, RI	*p*-Cymene	14.4
1021	1024	MS, RI	Limonene	0.4
1050	1054	MS, RI	γ-Terpinene	0.3
1114	1119	MS, RI	*E*-*p*-Mentha-2,8-dien-1-ol	0.2
1145	1148	MS, RI	Citronellal	0.1
1175	1178	MS, RI	Naphthalene	0.1
1235	1234	MS, RI	**Ascaridole**	**41.9**
1247	1252	MS, RI	*E*-Piperitone epoxide	1.1
1288	1289	MS, RI	Thymol	0.4
1299	1299	MS, RI	**Isoascaridole**	**7.5**
1347	1349	MS, RI	Thymol acetate	0.2
1477	1477	MS, RI	*E*-β-Ionone	0.1
1581		MS	3-Tetradecanone	0.4
			Yield	1.3
			Total identified	83.6

^a^KI_exp_ = retention indices are determined using n-alkanes (C_7_-C_17_).

^b^KI_lit_ = retention indices of reference compounds from literature.

^c^ID = Identification methods; MS = comparison of the mass spectrum with those of the computer mass libraries, and Adams (2007); RI = comparison of calculated RI with those reported in the literature.

^d^Compounds are listed in order of their retention time; the names and the percentages of main compounds are indicated in bold.

#### C. citratus

The oil yield of *C. citratus* was 1.7% (w/w). This value is higher than the one obtained with *C. citratus* (1.3%) collected in northern Brazil [49]. Essential oil of *C. citratus* was characterized by myrcene (12.4%), neral (33.1%) and geranial (44.3%). This result corroborates with previous results [49], which showed that the aerial part of *C. citratus* contains myrcene (10.7%), neral (30.8%) and geranial (53.9%). Furthermore, myrcene, neral and geranial were also the main compounds identified in *C. citratus* characterized in Burkina Faso, Brazil and Portugal [50-52].

#### C. giganteus

The oil yield of *C. giganteus* was 1.4% (w/w) and the main constituents of its essential oil were limonene (9.6%) and a set of monoterpene alcohols: *E*-*p*-mentha-1(7),8-dien-2-ol (19.6%), *E*-*p*-mentha-2,8-dienol (19.3%), *Z*-*p*-mentha-2,8-dienol (10.2%), *Z*-*p*-mentha-1(7),8-dien-2-ol (2.1%), *Z*-carveol (17.0%) and *E*-carveol (6.0%) together with *p*-menth-6-en-2,3-diol (3.2%) and carvone (3.2%).

This composition is similar to that of *C. giganteus* from Burkina Faso which was characterized by *E*-*p*-mentha-1(7),8-dien-2-ol, *E*-*p*-mentha-2,8-dienol, *Z*-*p*-mentha-2,8-dienol and *Z*-*p*-mentha-1(7),8-dien-2-ol [52] and from many West and Central African countries [28,53-55].

#### C. schoenanthus

The yield of *C. schoenanthus* essential oil was 2.6% (w/w). This result is similar to Ketoh *et al.*[56]. In the current work the major constituents were δ-2-carene (15.5%) and piperitone (58.9%); these results are consistent with the results obtained by Koba *et al*. and Ketoh *et al*. [38,56,57].

#### E. citriodora

Essential oil from *E. citriodora* was extracted with a yield of 4.6%. The main compounds detected by GC-MS were citronellal (52.8%), citronellol (20.0%), citronellyl acetate (9.0%) and neo-isopulegol (7.8%). Components like citronellal, citronellol and isopulegol were also detected in *E. citriodora* essential oil analyzed in Colombia [27,41].

#### E. tereticornis

*E. tereticornis* essential oil was extracted with a yield of 1.0%. This yield is lower than the one obtained from leaves of *E. tereticornis* (3.4%) isolated in Nigeria [58]. In our study, this oil was characterized by the presence of *p*-cymene (16.7%), cryptone (11.4%), spathulenol (13.5%), caryophyllene oxide (14.2%). Furthermore, compounds such as 4-terpineol (4.4%), phellandral (4.2%), cumin aldehyde (3.1%), β-phellandrene (2.9%), 1,8-cineole (2.2%) and humulene epoxide II (2.2%) were detected in significant amounts. *p*-Cymene, β-phellandrene, 1,8-cineole, 4-terpineol, cryptone and spathulenol were also identified in the oil of *E. tereticornis* analyzed in Benin, by Alitonou *et al.*[59] but in different amounts. Other differences were that it did not contain cumin aldehyde, humulene epoxide II and phellandral, whereas α-phellandrene, bicyclogermacrene and α-, β- or γ- isomers of eudesmol were detected. The presence of these main compounds was also noticed in the oil extracted in Argentina [60,61]. In contrast, the major constituents of the fresh leaf oil analyzed in India and Ethiopia were α-pinene and 1,8-cineole [62,63], whereas the main compounds in the essential oil from Nigeria were α- and β-pinene [58], the one from Cuba were 1,8-cineole and *p*-cymene [64], and the one from Algeria contained α-pinene, 1,8-cineole, β-ocimene, allo-aromadendrene and 4-terpineol [65].

#### C. tinctorium

Essential oil yield of *C. tinctorium* was 0.2% (w/w) which is higher than the one obtained in Burkina Faso (0.10%) by Benoit-Vical *et al*. [66]. The essential oil extracted from *C. tinctorium* in the current study is dominated by 3-tetradecanone (48.3%). 3-hexadecanone (7.4%), 2-tridecanone (3.4%), cyclododecanone (7.8%), dodecyl acetate (2.0%), methyl tetradecanoate (2.3%) and 1-tetradecanol acetate (4.3%). This chemical composition is similar to the result obtained by Benoit-Vical *et al.*[66], in the tubercle essential oil which contained 3-tetradecanone (64.6%), 3-hexadecanone (5.6%), dodecyl acetate (4.6%), in addition to 3-hexadecenone (3.4%) and tetradecyl acetate (11.7%).

#### C. planchonii

The essential oil of *C. planchonii* was extracted with a yield of 0.20% (w/w) lower than in the tubercle from Burkina Faso (0.30%) [66]. In the current study the composition of this essential oil was dominated by 3-tetradecanone (24.7%), ethyl tetradecanoate (11.4%) and isoamyl dodecanoate (14.1%), accompanied by 2-tridecanone (6.8%), dodecyl acetate (4.9%), 2-pentadecanone (5.7%), n-tetradecanol (3.0%), 3-hexadecanone (2.0%) and 4-octadecanone (2.2%). In the sample from Burkina Faso, 3-tetradecanone (69.8%) was the main compound, followed by tetradecyl acetate (14.4%), dodecyl acetate (4.7%), 3-hexadecanone (2.5%) and n-tetradecanol (1.0%) [66]. The sample characterized by Ouattara *et al*. [67] was similar to the previous one with major components involving 3-tetradecanone, 3-tetradecenone, dodecyl acetate, tetradecyl acetate, 2-tridecanone and β-elemene.

#### S. longepedunculata

The essential oil of *S. longepedunculata* was extracted with a yield of 0.7% (w/w). This yield is higher than the result obtained (0.30%-0.52%) by Alitonou *et al*. [68] in Benin and Adebayo *et al*. in Nigeria [69]. The *S. longepedunculata* essential oil was characterized by only one major constituent, namely methyl salicylate (99.4%). Indeed Nebie *et al*. [70] has shown that the essential oil of *S. longepedunculata* from Burkina Faso contains only one compound which is methyl salicylate. The same compound was also found in the methanol extract of *S. longepedunculata* from Ghana [71] and in essential oil extracted in Nigeria and Benin [68,69].

#### C. ambrosioides

*C. ambrosioides* essential oil was extracted with a yield of 1.3% (w/w). Its essential oil contained mainly ascaridole (41.9%). Some other components involving α-terpinene (16.5%), *p*-cymene (14.4%) and isoascaridole (7.5%) were identified as well. This chemical composition is similar to the one of a sample from China [39] but very different from a sample analyzed in India whose major components were m-cymene (43.9%) and myrtenol (13.3%) [72].

### Adult bioassay on susceptible strains of *Anopheles gambiae*

The resistant status of mosquito samples was determined according to the WHO criteria summarized as follows [42]:

- 98-100% mortality indicates susceptibility of the mosquito strain to the tested essential oil

- Mortality less than 98% is suggestive of the existence of a resistance to the essential oil that needs to be confirmed by two additional tests

- Mortality less than 90% suggests resistance in the mosquito population

#### KDT_50_ and KDT_95_

KDT_50_ and KDT_95_ calculated with 95% confidence limits are summarized in Table 7. The lowest KDT_50_ and KDT_95_ values were obtained with *C. citratus* and are respectively 2.1 min and 13.9 min at 0.5% and 1.2 min, 6.6 min at 1% whereas for permethrin (0.75%) these values were 11.3 min and 21.6 min. The highest KDT_50_ and KDT_95_ values were recorded with *C. planchonii* at 8% and were 11.3 min and 20.6 min, respectively.

**Table 7:** **Knock down times (KDT), mortality and susceptibility of essential oils on sensitive *Anopheles gambiae *** **“Kisumu”**

**Doses**	**0.25%**				**0.50%**				**1%**			
**Essential oils and controls**	**KDT**_**50**_^**a **^**(min)**	**KDT**_**95**_^**a **^**(min)**	**Mortality (%)**	**Susceptibility**^**b**^	**KDT**_**50**_^**a **^**(min)**	**KDT**_**95**_^**a **^**(min)**	**Mortality (%)**	**Susceptibility**^**b**^	**KDT**_**50**_^**a **^**(min)**	**KDT**_**95**_^**a **^**(min)**	**Mortality (%)**	**Susceptibility**^**b**^
***Cymbopogon citratus***	62.0	119.8	55.6	R	2.1	13.9	100	S	1.2	6.6	100	S
***Cymbopogon giganteus***	-	-	10.7	R	204.4	366.5	10.0	R	33.5	100.2	29.6	R
***Cymbopogon schoenanthus***	-	-	0.0	R	263.5	440.7	6.4	R	49.0	117.8	6.7	R
***Eucalyptus citriodora***	-	-	4.3	R	125.2	207.3	10.4	R	32.2	61.7	75.5	R
***Eucalyptus tereticornis***	12.4	49.8	72.5	R	5.4	18.2	86.7	R	2.5	16.1	98	S
***Cochlospermum tinctorium***	44.3	115.6	23.3	R	10.6	19.4	44.0	R	8.1	16.7	72.7	R
***Cochlospermum planchonii***	34.5	74.1	13.0	R	25.1	52.8	23.6	R	20.9	39.9	24.5	R
***Securidaca longepedunculata***	-	-	5.9	R	314.1	539.5	5.6	R	76.2	91.3	8.6	R
***Chenopodium ambrosioides***	736.3	1290.0	9.8	R	127.8	201.3	18.8	R	110.9	178.9	41.9	R
**Permethrin 0.75%**	11.3	21.6	100	S	11.3	21.6	100	S	11.3	21.6	100	S
**Negative control**	0	0	0	-	0	0	0	-	0	0	0	-
**Doses**	**2%**				**4%**				**8%**			
**Essential oils and controls**	**KDT**_**50**_^**a **^**(min)**	**KDT**_**95**_^**a **^**(min)**	**Mortality (%)**	**Susceptibility**^**b**^	**KDT**_**50**_^**a **^**(min)**	**KDT**_**95**_^**a **^**(min)**	**Mortality (%)**	**Susceptibility**^**b**^	**KDT**_**50**_^**a **^**(min)**	**KDT**_**95**_^**a **^**(min)**	**Mortality (%)**	**Susceptibility**^**b**^
***Cymbopogon citratus***	-	3.8	100	S	-	3.8	100	S	-	-	-	-
***Cymbopogon giganteus***	6.2	9.5	62.7	R	2.6	3.8	100	S	-	-	-	-
***Cymbopogon schoenanthus***	6.9	14.5	83.0	R	2.6	3.8	100	S	-	-	-	-
***Eucalyptus citriodora***	4.5	9.0	100	S	2.6	3.8	100	S	-	-	-	-
***Eucalyptus tereticornis***	-	3.8	100	S	-	3.3	100	S	-	-	-	-
***Cochlospermum tinctorium***	6.9	12.3	78.1	R	5.2	9.1	79.2	R	-	-	-	-
***Cochlospermum planchonii***	16.3	31.3	58.5	R	12.1	21.1	98.3	S	11.3	20.6	100	S
***Securidaca longepedunculata***	10.9	81.0	29.6	R	2.6	3.8	98.2	S	2.6	3.8	100	S
***Chenopodium ambrosioides***	12.8	25.2	100	S	2.6	3.8	100	S	-	-	-	-
**Permethrin 0.75%**	11.3	21.6	100	S	11.3	21.6	100	S	11.3	21.6	100	S
**Negative control**	0	0	0	-	0	0	0	-	0	0	0	-

^a^The KDT (KDT_50_ and KDT_95_) values were expressed with 95% confidence limits.

^b^S = Susceptible defined as 98-100% of mortality; RS = Resistance suspected defined as 90-97% of mortality; R = Resistance defined as <90% of mortality.

#### Mortality rates

Mortality rates to different essential oils are shown in Table 7. At 0.25%, the mortality rate of *A. gambiae* “*Kisumu*” varies from 0.0% to 72.5%. However, the mortality rates increased with the dosage. At 0.50%, mortality has reached 100% for *C. citratus,* whereas it was at 5.6% for *S. longepedunculata.* At 1%, mortality was still 100% for *C. citratus* whereas it was 6.7% for *C. schoenanthus*. At 2%, the mortality rate was 29.6% for *S. longepedunculata* and 100% for *C. citratus, E. citriodora, E. tereticornis* and *C. ambrosioides.* At 4% and 8%, the mortality rates varied from 79.2% to 100% for *C. tinctorium* and *C. planchonii.* To summarize these results, the susceptibility tests on the sensitive strain *A. gambiae* “*kisumu*” have demonstrated its susceptibility status on essential oils tested. The most efficient essential oil was *C. citratus* at 0.50%, followed by *E. tereticornis* at 1%, *E. citriodora* and *C. ambrosioides* at 2%, *C. schoenanthus, C. giganteus, C. planchonii* and *S. longepedunculata* at 4%.

#### Diagnostic concentrations

The diagnostic concentration is defined as twice the lethal concentration (LC) for 99% mortality (LC_99_) on sensitive strains [42]. Lethal concentration for 50% mortality (LC_50_), lethal concentration for 99% mortality (LC_99_) expressed with 95% confidence limits and diagnostic concentrations for all essential oils tested are summarized in Table 8.

**Table 8:** **LC**_**50**_**, LC**_**99 **_**and diagnostic concentration for all essential oils tested**

**Essential oils**	**LC**_**50**_^**a**^	**LC**_**99**_^**a**^	**Diagnostic concentration**	**Diagnostic concentration**	**Diagnostic concentration**
	**%**	**%**	**%**	**mg/ml**	**mg/cm**^**2**^
***Cymbopogon citratus***	0.237	0.386	0.77	7.7	0.085
***Cymbopogon giganteus***	1.600	3.682	7.36	73.6	0.82
***Cymbopogon schoenanthus***	1.570	2.739	5.48	54.8	0.60
***Eucalyptus citriodora***	0.900	1.685	3.37	33.7	0.37
***Eucalyptus tereticornis***	0.148	1.401	2.80	28.0	0.31
***Cochlospermum tinctorium***	1.16	5.781	11.56	115.6	1.28
***Cochlospermum planchonii***	2.314	7.608	15.22	152.2	1.69
***Securidaca longepedunculata***	2.489	4.919	9.84	98.4	1.09
***Chenopodium ambrosioides***	0.997	2.131	4.26	42.6	0.47

LC_50_: lethal concentration for 50% mortality; LC_99_: lethal concentration for 99% mortality.

^a^The lethal doses (LC_50_ and LC_95_) values were expressed with 95% confidence limits.

The lowest diagnostic concentration of 0.77% for *C. citratus* was not significantly different from the diagnostic dose of permethrin (0.75%). Other interesting values were also obtained for *E. tereticornis* (2.80%), *E. citriodora* (3.37%), and *C. ambrosioides* (4.26%). These plant species were followed by *C. schoenanthus* and *C. giganteus* whose diagnostic concentrations were 5.48% and 7.36%, respectively. The highest diagnostic doses were obtained with *S. longepedunculata* (9.84%), *C. tinctorium* (11.56%) and *C. planchonii* (15.22%).

All diagnostic doses obtained above were tested on the resistant strain of *A. gambiae* and results obtained were summarized in Table 9. Concerning the diagnostic doses tested, the lowest KDT_50_ and KDT_95_ were observed with *C. schoenanthus* (2.58 min, 3.84 min), followed by *S. longepedunculata, C. giganteus, E. tereticornis, C. ambrosioides* and *E. citriodora* for which the KDT_50_ and KDT_95_ were lower than 5 min and 6 min, respectively. A moderate knock down effect (KDT_50_ ≥ 12 min and KDT_95_ ≥ 20 min) was observed with *C. planchonii, C. tinctorium* and *C. citratus.*

**Table 9:** **KDT**_**50**_**, KDT**_**95 **_**and mortality of essential oils tested on the resistant strain of *Anopheles gambiae***

**Essential oils**	**Diagnostic doses**	**KDT**_**50**_^**a**^	**KDT**_**95**_^**a**^	**Mortality**	**Susceptibility**^**b**^
	**(%)**	**(min)**	**(min)**	**%**	
***Cymbopogon citratus***	0.77	15.77	26.00	100	S
***Cymbopogon giganteus***	7.36	2.92	5.53	100	S
***Cymbopogon schoenanthus***	5.48	2.58	3.84	100	S
***Eucalyptus citriodora***	3.37	4.02	5.93	100	S
***Eucalyptus tereticornis***	2.80	3.56	5.27	100	S
***Cochlospermum tinctorium***	11.56	12.01	20.67	90.4	RS
***Cochlospermum planchonii***	15.22	11.35	30.72	100	S
***Securidaca longepedunculata***	9.84	2.87	4.47	94.8	RS
***Chenopodium ambrosioides***	4.26	3.96	5.84	98.0	S
**Permethrin 0.75%**	0.75	90.87	145.37	62.3	R

KDT_50_: 50% knock down in mosquito’s population; KDT_95_: 95% knock down in mosquito’s population.

^a^The KDT (KDT_50_ and KDT_95_) values were expressed with 95% confidence limits.

^b^S = Susceptible defined as 98-100% of mortality; RS = Resistance suspected defined as 90-97% of mortality; R = Resistance defined as <90% of mortality.

The KDT_50_ observed was 6 to 35 fold lower than for permethrin, the positive control, and the KDT_95_ was 5 to 38 fold lower than permethrin. This observation demonstrates the promising insecticidal properties of these plants species on the *A. gambiae* resistant strain used.

Apart from the essential oil from *C. tinctorium* and *S. longepedunculata* for which resistance was suspected because the mortality was less than 97%, the resistant strain of *A. gambiae* was susceptible to all essential oils at diagnostic doses tested (Table 9). The resistance to permethrin in southern Benin has been demonstrated and was explained by the massive use of DDT in house spraying and agriculture, during the WHO malaria eradication program, which has permitted the apparition and the increase of *kdr* mutation in *A. gambiae* populations in Benin [7,10].

We have noticed that the knock-down times, the mortalities, and the resistance status of *A. gambiae* did not only depend on the values of doses used but mainly on the chemical composition of essential oils used.

Larvicidal activity of *C. citratus* essential oil on *Aedes aegypti* at lower concentrations (LC_50_ = 0.28 μl/ml and LC_90_ = 0.56 μl/ml) and its major component citral (neral and geranial) has been demonstrated by Freitas *et al.*[73]. *C. citratus* has also demonstrated a very good repellency against *A. aegypti*[30]. *C. citratus* has shown in the current study the best insecticidal activity against *A. gambiae*, but its KDT_50_ and KDT_90_ were higher than some of the other essential oils. The same conclusion has been found by Phasomkusolsil *et al*. [33] when the essential oil was tested on *A. aegypti, C. quinquefasciatus* and *Anopheles dirus.* The topical application of *C. citratus* showed high toxicity against *Sitophilus oryzae*[50]. *C. citratus* essential oil and citral have been shown to be potential anti-*Leishmania* agents [51]. Insecticidal and larvicidal activities of *C. citratus* have been attributed to citral that has demonstrated 100% mortality against *A. aegypti*, at 2.5 μl/ml with LC_50_ = 0.02 μl/ml and LC_90_ = 0.28 μl/ml respectively [73], and its repellent effect at 15% (v/v) is comparable to 5% *C. citratus* essential oil [74]. In conclusion, the presence of geranial and neral is potentially responsible for the insecticidal activity of the essential oil of *C. citratus,* as demonstrated in the current work.

*C. giganteus* essential oil, rich in limonene and (*Z* and *E*)-*p*-mentha-1(7),8-dien-2-ol such as the current sample from Benin, has proven to be toxic by fumigation to *Callosobruchus* species [28]. The insecticidal properties noticed in this study against *A. gambiae* might also be attributed to these main compounds.

Several studies on essential oils, rich in piperitone such as the essential oil from *C. schoenanthus* have demonstrated insecticidal activity against some pests. This is the case for *Cymbopogon olivieri,* which demonstrated good larvicidal activity against *A. stephensi* with LD_50_ = 321.9 mg/l [75]. Exposure of *Callosobruchus maculatus* to *C. schoenanthus* essential oil for 24 hours resulted in 90% of adult mortality at 6.7 μl/l [56]. Piperitone has been reported to be powerful against ants of *Crematogaster spp*[76]. Adults, newly laid eggs and neonate larvae of *C. maculatus* with an LC_50_ recorded at 1.6 ± 0.14 μl/l and all eggs were aborted at 6.7 μl/l with a total inhibition of the neonate larvae penetration in the seed [38]*.* Also in 2008, piperitone isolated from *Artemisia judaica* L., was studied against the third larvae of *Spodoptera littoralis* (Boisd) and has revealed a high insecticidal and antifeedant activity against this pathogen, with a LD_50_ = 0.68 μg/larvae [77]. Following this previous research we could attribute the insecticidal activity of *C. schoenanthus* to its main compound, i.e. piperitone.

The essential oil from *E. citriodora,* rich in citronellal, citronellol and isopulegol, has been revealed to be repellent against *Tribolium castaneum* at 0.084 ml/l and was more active than the commercial product IR3535 at 0.686 ml/l [41]. The insecticidal activity of *E. citriodora* has been demonstrated at 5 mg/ml against *Lutzomyia longipalpis*[27]. *E. citriodora* has also demonstrated larvicidal activity against *C. quinquefasciatus*[36] and acaricidal activity against larvae of *Amblyomma cajennense* and *Anocentor nitens*[78]. The presence of citronellal, citronellol and isopulegol could well explain the insecticidal activity of *E. citriodora* against *A. gambiae.*

The essential oil from *E. tereticornis* leaf extract has shown a larvicidal activity against *A. stephensi* at 160 ppm which has provoked 100% of oviposition deterrence [35]. The sensitivity of adults of *A. aegypti* has been shown, resulting from the presence of 1,8-cineole, α-pinene and *p*-cymene and is correlated to the amount of 1,8-cineole in the extract [26,60]. The insecticidal activity of the essential oil of *E. tereticornis* observed in the current work, might be explained by the presence of one of its major components (*p*-cymene) but also by a minor compound (1,8-cineole), which both have demonstrated insecticidal activity.

In essential oils from *Cochlospermum* species one minor compound (2-tridecanone) has been found to have insect repellent properties. Indeed, 2-tridecanone has demonstrated repellent activity against the granary weevil *Sitophilus granarius* and *S. zeamais* at 100 ppm and 500 ppm on wheat [79]. Its repellent activity was confirmed against ticks since 0.63 mg/cm^2^ was repellent to 87% of *Amblyomma americanum* after 12 hours and to 72% of *Dermacentor variabilis* after 15 hours [80]. The weak insecticidal activity of the essential oils of these two *Cochlospermum* species could be due to the low abundancy of 2-tridecanone.

Root powder, the methanol extract, and the main volatile component of *S. longepedunculata* (methyl salicylate) have proven to exhibit repellent and toxic effects against *S. zeamais*. In the same study, methyl salicylate has demonstrated a dose dependent fumigant effect with an LD_100_ of 60 μl in a 1-l container after 24 hours exposure on *S. zeamais*, *Rhyzopertha dominica* and *Prostephanus truncates* and after 6 days exposure, 100% mortality could be recorded with 30 μl in a 1-l container [31].

The *C. ambrosioides* essential oil has demonstrated a larvicidal activity against *A. arabiensis* and *A. aegypti* after 24 hours exposure with LC_50_ and LC_90_ equal to 17.5 ppm and 33.2 ppm for *A. arabiensis* and 9.1 ppm and 14.3 ppm for *A. aegypti* under laboratory conditions [81]. Contact and fumigant toxicity of isolated compounds from this plant species have shown that ascaridole (LC_50_ = 0.84 mg/l) followed by isoascaridole (LC_50_ = 2.45 mg/l) were the most efficient insecticidal compounds by fumigation and contact with LC_50_ = 0.86 mg/l (ascaridole) and 2.16 mg/l (isoascaridole). The crude oil was less active with LC_50_ = 3.08 mg/l by fumigation and 2.12 mg/l by contact [39]. The insecticidal activity of *C. ambrosioides,* noticed in the study, might be explained by the presence of ascaridole and isoascaridole, which were among its major constituents.

## Conclusions

The current study has dealt with the insecticidal properties of essential oils of nine plant species traditionally used in Benin for their repellency against *A. gambiae* bites. This research has shown that the essential oils from all the plant species studied, have insecticidal properties against this vector of malaria. The most promising was *C. citratus* followed in order of effectiveness by *E. tereticornis, E. citriodora, C. ambrosioides*, *C. schoenanthus, C. giganteus* and *C. planchonii.* The chemical composition of each plant essential oil has been elucidated by GC-MS and correlated with the insecticidal properties of these plant species. To our knowledge, it was the first time that diagnostic doses of essential oils on *A. gambiae* were determined, using the WHO susceptibility test protocol. These doses were presented in %, mg/ml and mg/cm^2^ to facilitate further research on these plant species. KDT_50_ and KDT_95_, LC_50_ and LC_99_ and results obtained have proven that all essential oils from these plant species are more effective against the resistant strain of *A. gambiae* than permethrin at the diagnostic doses tested. *C. citratus, E. tereticornis, E. citriodora* and *C. ambrosioides* as well as essential oil isolated components, such as citral, piperitone, 1,8-cineole, citronellal, 2-tridecanone, methyl salicylate, which possess demonstrated insecticidal properties, may be included in malaria vector control programs. These plants, occurring in the natural environment of local populations, could be obtained at lower cost and represent today a valuable source of bioactive compounds for the protection of the population against malaria.

## Competing interests

The authors declare that they have no competing interests.

## Authors’ contributions

ADB, FA, DCKS, SM, NDK and MCA have defined the study. HPY has conducted the identification and the harvesting of the plant species. ADB and PMB have performed the experiments and interpretation of data. SM, NDK, DCKS, FA, PMB, MCA and ADB drafted and revised the manuscript. All authors read and approved the final version of the manuscript.

## Aknowledgment

We are grateful to the team of the Centre de Recherche en Entomologie de Cotonou, for the realization of insecticidal tests on *A. gambiae*, as well as to the Belgian Technical Cooperation, Ghent University and the Research Foundation-Flanders for financial support of this research.
